# Utility of self-rated adherence for monitoring dietary and physical activity compliance and assessment of participant feedback of the Healthy Diet and Lifestyle Study pilot

**DOI:** 10.1186/s40814-021-00786-3

**Published:** 2021-02-11

**Authors:** Holly O’Reilly, Chloe E. Panizza, Unhee Lim, Kim M. Yonemori, Lynne R. Wilkens, Yurii B. Shvetsov, Michelle N. Harvie, John Shepherd, Fengqing Maggie Zhu, Loïc Le Marchand, Carol J. Boushey, Kevin D. Cassel

**Affiliations:** 1 The Technological University Dublin and The University of Dublin, Trinity College, 191 North Circular Road, D07 EWV4 Dublin, Ireland; 2 University of Hawai’i Cancer Center, University of Hawaii at Manoa, 701 Ilalo Street, Honolulu, HI 96813 USA; 3 Manchester University Hospital Foundation NHS Trust, Cobbett House, Oxford Road, Manchester, M13 9WL UK; 4 Purdue University, 610 Purdue Mall, West Lafayette, IN 47907 USA

**Keywords:** Pilot study, Qualitative assessment, Randomized controlled trial, Self-rated adherence, Visceral adipose tissue, Weight loss

## Abstract

**Background:**

We examined the utility of self-rated adherence to dietary and physical activity (PA) prescriptions as a method to monitor intervention compliance and facilitate goal setting during the Healthy Diet and Lifestyle Study (HDLS). In addition, we assessed participants’ feedback of HDLS. HDLS is a randomized pilot intervention that compared the effect of intermittent energy restriction combined with a Mediterranean diet (IER + MED) to a Dietary Approaches to Stop Hypertension (DASH) diet, with matching PA regimens, for reducing visceral adipose tissue area (VAT).

**Methods:**

Analyses included the 59 (98%) participants who completed at least 1 week of HDLS. Dietary and PA adherence scores were collected 8 times across 12 weeks, using a 0–10 scale (0 = not at all, 4 = somewhat, and 10 = following the plan very well). Adherence scores for each participant were averaged and assigned to high and low adherence categories using the group median (7.3 for diet, 7.1 for PA). Mean changes in VAT and weight from baseline to 12 weeks are reported by adherence level, overall and by randomization arm. Participants’ feedback at completion and 6 months post-intervention were examined.

**Results:**

Mean ± SE, dietary adherence was 6.0 ± 0.2 and 8.2 ± 0.1, for the low and high adherence groups, respectively. For PA adherence, mean scores were 5.9 ± 0.2 and 8.5 ± 0.2, respectively. Compared to participants with low dietary adherence, those with high adherence lost significantly more VAT (22.9 ± 3.7 cm^2^ vs. 11.7 ± 3.9 cm^2^ [95% CI, − 22.1 to − 0.3]) and weight at week 12 (5.4 ± 0.8 kg vs. 3.5 ± 0.6 kg [95% CI, − 3.8 to − 0.0]). For PA, compared to participants with low adherence, those with high adherence lost significantly more VAT (22.3 ± 3.7 cm^2^ vs. 11.6 ± 3.6 cm^2^ [95% CI, − 20.7 to − 0.8]). Participants’ qualitative feedback of HDLS was positive and the most common response, on how to improve the study, was to provide cooking classes.

**Conclusions:**

Results support the use of self-rated adherence as an effective method to monitor dietary and PA compliance and facilitate participant goal setting. Study strategies were found to be effective with promoting compliance to intervention prescriptions.

**Trial registration:**

ClinicalTrials.gov Identifier: NCT03639350. Registered 21st August 2018—retrospectively registered.

**Supplementary Information:**

The online version contains supplementary material available at 10.1186/s40814-021-00786-3.

## Key messages regarding feasibility

1. What uncertainties existed regarding the feasibility?

- The utility of self-rated adherence to monitor participant compliance to dietary and physical activity prescriptions and to guide motivational interviewing.
- The acceptability of intervention strategies used to promote participant adherence. A key study strategy included adapting the dietary educational materials originally developed and tested for use among women in Greater Manchester, UK, for use with East Asian Americans living in Hawaii.
- Integrating dietary educational materials with motivational interviewing techniques which were predominantly delivered over the telephone by study dietitians.

2) What are the key feasibility findings?

Our results support the use of self-rated adherence to dietary and physical activity prescriptions as an effective method to monitor compliance and facilitate participant goal setting. The study strategies used in HDLS were found to be effective with promoting compliance to dietary and physical activity prescriptions.

3) What are the implications of the feasibility findings for the design of the main study?

Based on the results of the HDLS pilot, self-rated adherence to dietary and physical activity prescriptions, support from study dietitians mostly delivered via telephone, and culturally adapted dietary assessment materials are important study strategies to implement in the main HDLS intervention to ensure participant engagement and compliance. Feedback from participants suggested the incorporation of cooking classes and demonstrations into future trials may further complement dietary adherence. Cooking classes and demonstrations were not included in the current pilot study; therefore, require further investigation.

## Background

Overweight and obesity are pervasive risk factors for many non-communicable diseases [1]. In particular, excess visceral adipose tissue (VAT) is associated with increased risk of cardio-metabolic disease, coronary artery calcification, type 2 diabetes, metabolic syndrome, certain cancers, and non-alcoholic fatty liver disease [2–7]. Adherence to lifestyle modification programs is known to be difficult [8, 9]; however, greater adherence is associated with improved obesity outcomes [9–14]. No known study has assessed the association between self-rated intervention adherence and VAT loss.

Research on lifestyle interventions aimed at reducing VAT have primarily been quantitative in nature [15–19]. However, a mixed-methods approach, using both quantitative and qualitative data, would help provide a more complete picture of study effectiveness, including participants’ perspectives [20]. This is especially important in nutrition interventions aimed at changing behaviors [20], and may assist in identifying factors influencing study adherence.

Previously, our team reported the quantitative results of the randomized Healthy Diet and Lifestyle study (HDLS) pilot [21], aimed at reducing VAT among East Asian American adults. VAT and weight decreased in both study arms, but significantly more in the intermittent energy restriction combined with a Mediterranean diet (IER + MED) than the Dietary Approaches to Stop Hypertension (DASH) diet group at 12 weeks [21]. The current analysis aims to assess the utility of a self-rated scale used to monitor participants’ dietary and physical activity compliance and which facilitated goal setting using motivational interviewing principles during the intervention sessions. We also aim to evaluate the HDLS pilot by analyzing participants’ feedback collected upon completion and at 6-months post intervention. Results will help inform the feasibility and study design of the larger main HDLS intervention.

## Methods

### Study design

The HDLS pilot study was a 12-week randomized trial conducted at the University of Hawaii Cancer Center (UHCC) between September 2016 and October 2017. Extensive details of the intervention are provided in a previous publication [21]. Briefly, eligibility included being of East Asian ancestry (Japanese, Chinese, or Korean), residing in Honolulu County, BMI between 25 and 40 kg/m^2^, ages 35 to 55 years, no pregnancy, and no serious health issues (including issues that would limit the ability to meet the physical activity prescriptions). Inclusion criteria included normal blood count and biochemistry profile and whole-body dual-energy X-ray absorptiometry (DXA)-derived VAT at L4–L5 ≥ 90 cm^2^ for men and ≥ 80 cm^2^ for women as determined at the eligibility clinic visit. As part of the screening for enrollment participants completed a Physical Activity Readiness Questionnaire [22, 23]. However, physical activity was not objectively assessed and there were no specific inclusion criteria for baseline physical activity levels. The primary outcome of HDLS focused on dietary exposures, thus the decision to prescribe the same physical activity prescription to each study arm to reduce any possible confounding. Throughout the intervention, participants were encouraged to meet and to not exceed the physical activity prescriptions. The enrollment goal was to recruit 70 persons to achieve a final sample of 50 persons to account for an attrition rate of ~ 23%, as reported in past studies [15].

HDLS included baseline and week 12 measurements of anthropometry and DXA [21]. For the current analysis, outcomes of interest include body weight and VAT. One participant was excluded from analyses as he/she dropped out during the first week of HDLS and, thus, had no self-rated adherence data.

**Fig. 1:**
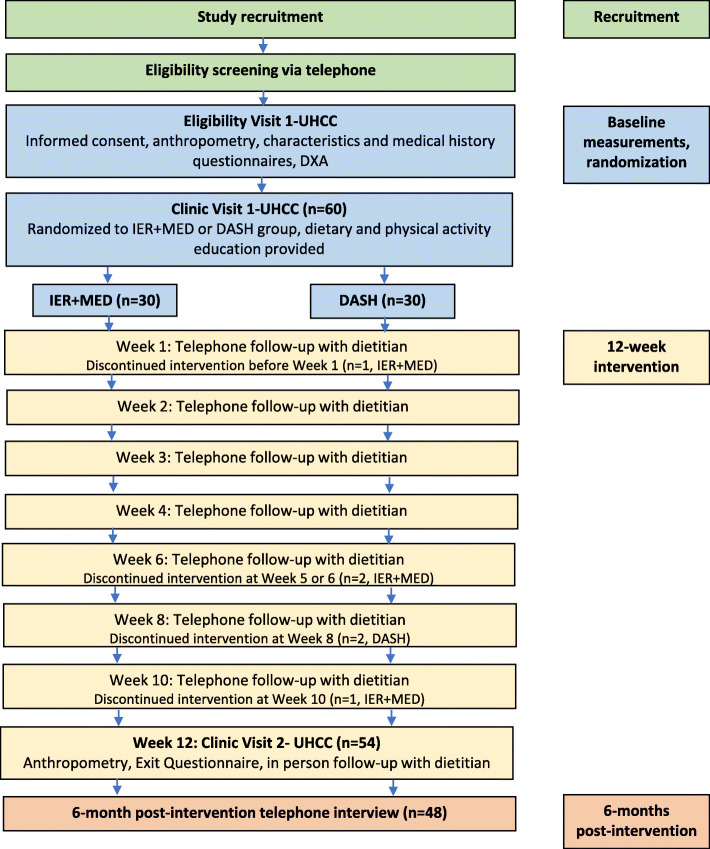
Healthy Diet and Lifestyle Study flow diagram

The study protocol [NCT03639350] was approved by the institutional review board at the University of Hawaii at Manoa. Study volunteers provided written informed consent. Figure 1 is a CONSORT diagram and the CONSORT checklist is provided as supplementary material (Additional file 1)

### Randomization and masking

Once eligibility was confirmed, stratified and blocked randomization was used to allot participants into either the IER+MED (*n* = 30) or DASH (*n* = 30) group, to ensure balance between groups. Randomization strata were defined by sex and high (80 cm^2^ for women or 90 cm^2^ for men to < 150 cm^2^) or very high (≥ 150 cm^2^) VAT. The assigned diets were labeled as either Diet A or Diet 1 to blind participants, recruitment, and clinic staff to IER + MED or DASH group allocation. Research dietitians, who were responsible for intervention activities, were blinded to participant measures except diet and body weight.

### Procedures and outcomes

#### Dietary and physical activity prescriptions

Diet and physical activity prescriptions in HDLS have been reported previously [21]. Briefly, the intervention group was assigned an IER + MED diet. The active comparator group was assigned a euenergetic (met estimated energy requirements (EER)) DASH diet for 12 weeks. Dietary materials for IER + MED were originally developed and tested for use among white women in the UK [15], and were modified to include examples of foods and beverages more readily available in Hawaii [21]. Both groups were advised to walk up to 1 h daily, 5 days per week, to reduce confounding due to physical activity. Participants were encouraged to choose walking as their physical activity; however, alternatively they could select another physical activity to meet their prescription. Participants in the IER + MED group were advised to exercise only on their MED days.

During an in-person dietary consultation (~ 45–60 min) participants were provided with a personalized, group-specific, diet booklet (depicting serving sizes within food groups and examples of foods), individualized food lists and menus, and trackers to encourage compliance to prescriptions. Dietitians called participants once a week between weeks 1 and 4, and at week 6, 8, and 10, and conducted an in-person follow-up at week 12, to assess participants’ adherence to their intervention plans [24] and provide guidance using motivational interviewing principles [25, 26].

### Assessment of adherence to prescribed diet and physical activity prescriptions

During follow-up calls conducted by study dietitians and the in-person dietitian consultation at week 12, all participants were asked: “How well have you been following your diet plan? On a scale of zero to ten with zero being not at all, four being somewhat, and ten being following the plan very well, where would you place yourself?”. Similarly, the same questions were asked for adherence to physical activity prescriptions. Self-rated adherence scores assisted with identifying participant’s barriers to change and setting personal diet and physical activity goals to achieve by the next follow-up call [25, 27].

These assessment questions were adapted from those used to assess motivation and confidence to change dietary behaviors used by Resnicow et al. [25, 26, 28]. For example, in the Body & Soul Study, participants were asked “on a scale of 1 to 10 (with 10 being the highest), how motivated or interested are you in increasing your fruit and vegetable consumption?” Resnicow et al. found that this time and cost-effective assessment technique assisted with increasing fruit and vegetable intake among African Americans in a church setting.

Responses to self-rated adherence scores across the 12-week HDLS were averaged for each person, and participants were divided into a high or low level of adherence, split by median score (7.3 for diet and 7.1 for physical activity).

### Participant feedback

During the clinic visit at week 12, participants completed a self-administered exit questionnaire. The two questions relevant to this analysis were if he/she would have been interested in cooking classes or demonstrations and whether the materials provided were sufficient to be able to follow the diet plan. Participants recorded their responses using a 5-point Likert scale (strongly agree/agree/neither agree or disagree/disagree/strongly disagree).

At 6 months post-intervention, recruitment staff performed a follow-up, by telephone, of participants who completed the HDLS. Participants were interviewed using a standardized questionnaire tailored to the study objectives. Questions pertinent to this analysis included “What type or types of exercise have you been doing since you completed the study?”; “Are you still trying to follow the diet you were assigned to during the study?”; “If no, can you describe the type of diet that you have been following since you completed the study?”; and “Do you have any comments or suggestions as to how the study could be improved?”.

### Statistical analysis

Analyses were limited to participants with complete week 1 study data (*n* = 59). Multiple imputation was used to replace missing values of the outcome and exposure variables and generate five imputed datasets. Missing data for participants who dropped out of the study after the week 1 follow-up call (*n* = 5) were also imputed. Less than 5% of data values were missing. Mean changes in VAT and weight loss, from baseline to 12 weeks, were computed by self-reported adherence level, overall and by randomization arm, and compared between adherence levels using a *t* test. Responses to 5-point Likert scale questions are reported as frequencies and percentages. Quantitative analyses were performed using IBM SPSS Statistics version 26 (IBM Corp., Armonk, NY, USA) and statistical significance was defined as *p* < 0.05.

Qualitative methods were used to evaluate responses to the open-ended 6-month post-intervention telephone interview questions [29]. Responses to open-ended questions from each participant were transcribed separately by staff members not involved in the study. Co-investigators (KC, HO) and another staff member independently coded each response transcript. Each coder identified and nominated common themes and preliminary codes, and all codes were reviewed and discussed by the team until a final consensus was reached. A codebook was then developed for analysis using NVIVO Version 11 (QSR International, Melbourne, Australia) and used to drive a subsequent thematic analysis of all interview transcript data 29.

## Results

### Characteristics

Of the 60 participants enrolled in HDLS, 6 (10%) dropped out (IER + MED: 4; DASH: 2), with only 1 participant withdrawing from the study before the week 1 follow-up call. Of the 54 participants completing HDLS, all 54 participants completed the Exit Questionnaire and 48 participants responded to the 6-month post-intervention telephone interview. Of participants included in the current analysis, 29 (48.2%) were randomized to IER + MED and 30 (50.8%) to DASH (Table 1). The study participants were mostly women (69.5%), participants with high VAT (67.8%), and of Japanese ancestry (62.7%). Baseline characteristics were similar for the 59 participants included in this analysis, the 54 participants who completed the week 12 visit and the 48 who completed the 6-month post-intervention survey.

**Table 1:** Baseline characteristics of participants (*n* = 59) by self-rated adherence to dietary and physical activity prescriptions

		Dietary adherence^a,b^		Physical activity adherence^b,c^	
Characteristic	All	Low	High	Low	High
Study arm, *n* (%)^d^					
IER+MED^e^	29 (49.2)	14 (48.3)	15 (51.7)	12 (41.4)	17 (58.6)
DASH^f^	30 (50.8)	17 (56.7)	13 (43.3)	16 (53.3)	14 (46.7)
Sex, *n* (%)					
Men	18 (30.5)	7 (38.9)	11 (61.1)	8 (44.4)	10 (55.6)
Women	41 (69.5)	24 (58.5)	17 (41.5)	20 (48.8)	21 (51.2)
Visceral adipose tissue, *n* (%)					
High^g^ (80 or 90 to < 150 cm^2^)	40 (67.8)	23 (57.5)	17 (42.5)	20 (50.0)	20 (50.0)
Very high (≥ 150 cm^2^)	19 (32.2)	8 (42.1)	11 (57.9)	8 (42.1)	11 (57.9)
Ethnicity, *n* (%)					
Chinese	8 (13.6)	4 (50.0)	4 (50.0)	2 (25.0)	6 (75.0)
Japanese	37 (62.7)	17 (45.9)	20 (54.1)	15 (40.5)	22 (59.5)
Korean	7 (11.9)	5 (71.4)	2(28.6)	7 (100.0)	0 (0.0)
Mixed Asian	7 (11.9)	5 (71.4)	2 (28.6)	4 (57.1)	3 (42.9)
Other characteristics, mean ± SD					
Age, years	47.4 ± 5.1	46.1 ± 5.3	48.9 ± 4.4	45.9 ± 5.7	48.9 ± 4.0
Weight, kg	80.4 ± 12.4	78.2 ± 11.4	82.8 ± 13.4	80.0 ± 11.3	80.7 ± 13.4
Body mass index, kg/m^2^	30.7 ± 3.4	30.2 ± 3.4	31.2 ± 3.3	30.5 ± 3.6	31.0 ± 3.1

^a^Self-rated dietary adherence ranging from zero being not at all to ten being following the plan very well, split by median adherence (7.3)

^b^Imputed values for missing adherence data

^c^Self-rated physical activity adherence ranging from zero being not at all to ten being following the plan very well, split by median adherence (7.1)

^d^Column percentages for overall column and row percentages for data by self-rated adherence level

^e^Intermittent energy restriction combined with a Mediterranean diet (IER + MED)

^f^Euenergetic Dietary Approaches to Stop Hypertension diet (DASH)

^g^Women at ≥ 80 cm^2^ and men at ≥ 90 cm^2^

Splitting data by median adherence, baseline characteristics were similar between dietary adherence groups and between physical activity adherence groups (Table 1). The largest differences in adherence were seen between ethnic groups. For dietary adherence, 71.4% (*n* = 5) participants of Korean or Mixed Asian ancestry were in the low category. For physical activity adherence, 100% (*n* = 7) of participants with Korean ancestry were in the low group, and 75% (*n* = 6) of participants of Chinese ancestry were in the high adherence group.

### Adherence

Overall, mean ± SE, dietary adherence over 12 weeks was 6.0 ± 0.2 and 8.2 ± 0.1, for the low and high adherence groups, respectively. Ranges of dietary adherence scores were 2.9–7.7 and 7.1–9.9, respectively. For physical activity adherence, mean scores were 5.9 ± 0.2 and 8.5 ± 0.2 for the low and high adherence groups, and ranged from 3.0–7.2 and 7.0–10.0, respectively.

Compared to participants with low self-rated adherence to dietary prescriptions, those with high adherence lost significantly more VAT (22.9 ± 3.7 cm^2^ vs. 11.7 ± 3.9 cm^2^ [95% CI*,* − 22.1 to − 0.3]) and weight at week 12 (5.4 ± 0.8 kg vs. 3.5 ± 0.6 kg [95% CI, − 3.8 to − 0.0]) (Table 2). For physical activity, compared to participants with low adherence, those with high adherence lost significantly more VAT (22.3 ± 3.7 cm^2^ vs. 11.6 ± 3.6 cm^2^ [95% CI, − 20.7 to − 0.8]) (Table 3). Weight loss was also greater for those with high vs. low adherence to physical activity prescriptions (5.0 ± 0.7 kg vs. 3.7 ± 0.7 kg); however, these differences were not significant (95% CI, − 3.2 to 0.6). Within study arm comparisons, high dietary adherence and high physical activity adherence had greater VAT and weight loss than their counterpart low adherence groups, but these differences were not significant (Tables 2 and 3). Repeating analyses using % change in VAT and weight instead of absolute change produced similar results. The association between continuous values for self-rated adherence and % change in VAT and weight were also examined. Results were similar to the primary analyses, with the exception of the association between self-rated physical activity adherence and % change in body weight which was found to be significant among all participants (95% CI, 0.0 to 1.3).

**Table 2:** Change in visceral adipose tissue (VAT) and weight by self-rated adherence to dietary prescriptions^a^

		VAT loss at week 12 (cm^2^) *n* = 59		Weight loss at week 12 (kg) *n* = 59	
	Self-rated adherence^b^	Mean (SE)	95% CI	Mean (SE)	95% CI
Total	Low	11.7 (3.9)	− 22.1 to − 0.3	3.5 (0.6)	− 3.8 to − 0.0
	High	22.9 (3.7)		5.4 (0.8)	
IER + MED^c^	Low	18.5 (6.7)	− 25.0 to 8.4	4.6 (1.0)	− 4.5 to 0.6
	High	26.8 (4.3)		6.6 (0.9)	
DASH^d^	Low	6.3 (3.9)	− 25.6 to 2.2	2.6 (0.7)	− 4.0 to 1.1
	High	18.0 (6.2)		4.1 (1.2)	

^a^Imputed values for missing adherence data

^b^Mean self-rated dietary adherence ranging from zero being not at all to ten being following the plan very well, split by median adherence (7.3)

^c^Intermittent energy restriction combined with a Mediterranean diet

^d^Euenergetic Dietary Approaches to Stop Hypertension diet

**Table 3:** Change in visceral adipose tissue (VAT) and weight by self-rated adherence to physical activity prescriptions^a^

		VAT loss at week 12 (cm^2^) *n* = 59		Weight loss at week 12 (kg) *n* = 59	
	Self-rated adherence^b^	Mean (SE)	95% CI	Mean (SE)	95% CI
Total	Low	11.6 (3.6)	− 20.7 to − 0.8	3.7 (0.7)	− 3.2 to 0.6
	High	22.3 (3.7)		5.0 (0.7)	
IER + MED^c^	Low	17.8 (5.9)	− 22.8 to 4.8	5.5 (1.1)	− 3.0 to 2.5
	High	26.8 (4.4)		5.8 (0.9)	
DASH^d^	Low	6.8 (4.1)	− 23.6 to 3.7	2.5 (0.8)	− 4.2 to 0.8
	High	16.8 (5.8)		4.2 (1.0)	

^a^Imputed values for missing adherence data

^b^Mean self-rated physical activity adherence ranging from zero being not at all to ten being following the plan very well, split by median adherence (7.1)

^c^Intermittent energy restriction combined with a Mediterranean diet

^d^Euenergetic Dietary Approaches to Stop Hypertension diet

### Participant feedback

Answering the question, “materials provided were sufficient to be able to follow the diet plan”, 44 (81%) participants rated that they strongly agree or agree, 8 (15%) that they neither agree nor disagree, and 2 (4%) participants did not respond. For the question “Would in-house classes or cooking demonstrations have been of interest?” 36 (67%) participants answered yes, 12 (22%) reported no, 2 (4%) were undecided, and 4 (7%) did not answer the question. Women tended to be more interested in the in-house classes or cooking demonstrations than men (75% and 47%, respectively).

The thematic structure identified for the open-ended questions as part of the 6-month post-intervention telephone interview followed the topics of the survey questions including (1) exercise; (2) diets; (3) comments; and (4) suggestions [29]. Participant responses were summarized based on this thematic structure.

#### Exercise

Almost all (*n* = 46, 96%) participants reported continuing exercise at 6 months post-intervention. Overall, respondents reported 60 types of exercise regimens they adopted or continued after the study, with 31 (65%) reporting “walking” as a primary form of exercise. Other popular exercise included running, swimming, paddling, tennis, golf, cycling, weightlifting, fishing, Zumba, Step Aerobics, Aqua Aerobics, high intensity interval training, calisthenics, stair climbing, and use of a gymnasium.

#### Diets

Approximately half of the respondents reported “*yes*”, they were still trying to follow their prescribed diet at 6 months post-intervention (IER + MED, 66.7%; DASH, 44.0%). Of those participants not following the diet at 6 months post-study, many (*n* = 8) reported focusing on a healthy diet without mentioning the specific dietary changes. For example, “Your study increased my awareness regarding foods and I am eating healthier, and use the basics of the study as a guide.” Also, although no longer following their prescribed diet, participants reported substituting nutrient poor items for nutrient dense choices (*n* = 4), reducing their portion size (*n =* 9), decreasing intake of sugar, meat, or carbohydrates (*n =* 7), and increasing intake of fruit and vegetables (*n =* 14). An example being, “gave up some snack foods and unhealthy food. More aware of healthier options.” In addition, participants reported adopting a modified version of the study diet with the re-adoption of non-study foods (*n* = 6), for example, “following protocol, but added dark chocolate.”

#### Comments

Under this theme, praise for the support of the intervention dietitians was the most frequent comment (*n* = 14). For example, “Helpful to have dietitian suggestions and accountability.” Unfamiliarity with the foods prescribed during the study, and preparation of these foods (*n* = 2) was the second most frequent comment.

#### Suggestions

The most common response, on how to improve the study, was to provide cooking education (*n* = 8) in the form of classes delivered either in-person or online; and the creation of a cookbook to support the study. For example, “*would really appreciate cooking classes*.” The Identification of more varied foods to support the study was also suggested, including the identification of premade foods (*n* = 3) that would be acceptable for use and longer study duration (*n* = 1).

## Discussion

Among all participants, higher self-rated adherence to dietary or physical activity prescriptions was associated with significantly greater loss of VAT on completion of the 12-week HDLS pilot. These results support the utility of self-rated adherence as a method for monitoring compliance and facilitating participant goal setting during interventions aimed at reducing VAT. The association found between dietary adherence and weight loss also supports the utility of this assessment method and aligns with previous findings, where higher self-rated adherence resulted in greater weight loss [10, 14].

The single-item questions used to assess adherence in HDLS were adapted from questions used by Resnicow et al. [25, 26]. Resnicow et al. demonstrated that these single-item questions, based on motivational interviewing, were effective at evaluating confidence and motivation to change, and for eliciting motivational messages and barriers to change [25, 26, 28]. Similarly, Dansinger et al. used a 0–10 scale to assess participants self-rated adherence and the effectiveness of 4 popular diets for weight loss [10]. In both the current analysis and the Dansigner et al. study, greater dietary adherence was associated with weight loss. Results from the HDLS pilot, and these previous studies, support the utility of single-item self-rated adherence questions to assess intervention compliance and assist with facilitating behavior change for the main HDLS intervention.

Several other studies have verified the agreement between self-evaluation and behavior. A cross-sectional study by Adjoian et al. assessed the validity of self-rated overall diet quality compared to Healthy Eating Index-2010 (HEI-2010) scores among a multiethnic adult population in New York City (NYC) [30]. Those with lower self-rated diet quality had significantly lower HEI-2010 scores. In regard to physical activity, a single-item questionnaire, assessing participants’ activity in the previous week, has been validated against accelerometer data [31]. The question asks “In the past week, on how many days have you done a total of 30 min or more of physical activity, which was enough to raise your breathing rate? This may include sport, exercise and brisk walking or cycling for recreation or to get to and from places, but should not include housework or physical activity that may be part of your job”. These studies add to the value of short self-report questionnaires, where participant responses matched direct objective measures of study outcomes.

Key strategies for dietary adherence include designing weight loss studies that are tailored to participants dietary preferences and promote self-monitoring of dietary intake [9]. These strategies were employed in HDLS [21], which may explain why mean dietary adherence for both study arms was relatively high (IER + MED, 7.3; DASH, 6.8), and participants’ positive reflections of the study. However, modifications to the design of HDLS may help to further improve dietary adherence. For example, responses to both the Exit Questionnaire and 6-month post-study telephone interview indicated that most participants (~ 2/3) were interested in cooking classes and demonstrations. The integration of these classes may help to further personalize dietary prescriptions, reduce any unfamiliarity of foods prescribed, increase participants’ confidence in preparing meals [32], and ultimately improve study compliance, long-term adherence, and adoption.

HDLS participants did not provide any comments or suggestions on how to improve physical activity prescriptions. However, ethnicity appeared to be a large driver for physical activity adherence, with all participants of Korean ancestry (*n* = 7) being in the low adherence category. A review on adherence to lifestyle modifications programs for weight management highlights strategies which may help improve physical activity compliance for those in the low adherence category [9]. The authors identified psychosocial factors, socio-demographic factors, behavioral factors, and physical factors as influencing adherence. For example, psychological factors influencing adherence include self-efficacy, depression, motivation, stress, body shape concern, quality of life, and stage of change. Socio-demographic factors include age, gender, employment status, and education [9]. Focusing on reasons for ethnic differences in physical activity adherence and additional predictors of adherence may help to improve physical activity compliance scores in future trials.

The strengths of HDLS include the evidence-based strategies implemented to ensure participant engagement and compliance. These include the adoption of dietary protocols by Harvie et al. [15], and behavior change strategies from Body and Soul [25, 26]. Being a pilot study, a limitation was the small sample size which may have reduced statistical power to show significant associations between adherence and changes in VAT and body weight by study arms. Also, the association seen between weight loss and adherence level may have been due to reverse causation. Participants with greater weight loss may have thought they were adhering to the dietary and physical activity prescriptions more closely, and rated their adherence higher. However, change in VAT is harder for participants to self-monitor and change in VAT between adherence arms was proportionately larger than change in weight. Therefore, it is unlikely VAT results were due to reverse causation. Another limitation is changes in VAT and weight were used as proxy measurements for adherence to intervention prescriptions. To assess the validity of self-rated compliance to monitor intervention adherence, future studies should compare self-rated adherence results to more direct objective measurements as opposed to proxy methods (e.g., compare self-rated physical activity adherence to accelerometer data).

## Conclusions

Results support the utility of self-rated compliance as a method to monitor dietary and physical activity adherence and facilitate participant goal setting. Overall, participant feedback on HDLS was positive, demonstrating the feasibility and acceptability of study strategies for use in the main HDLS intervention. The incorporation of cooking classes and demonstrations into future trials warrant investigation and may further complement dietary adherence.

## Supplementary Information

**Additional file 1.** CONSORT 2010 checklist of information to include when reporting a pilot or feasibility trial*.

## Data Availability

The datasets used and/or analyzed during the current study are available from the corresponding author on reasonable request.

## Abbreviations

BMI: Body mass index
DASH: Dietary Approaches to Stop Hypertension
DXA: Dual-energy X-ray absorptiometry
HDLS: Healthy Diet and Lifestyle Study
IER + MED: Intermittent energy restriction combined with a Mediterranean diet
UHCC: University of Hawaii Cancer Center
VAT: Visceral adipose tissue
